# Movement related dynamics of subthalmo-cortical alpha connectivity in Parkinson's disease

**DOI:** 10.1016/j.neuroimage.2012.12.041

**Published:** 2013-04-15

**Authors:** Ashwini Oswal, Peter Brown, Vladimir Litvak

**Affiliations:** a Wellcome Trust Centre for Neuroimaging, UCL Institute of Neurology, 12 Queen Square, London WC1N 3BG, UK; b Nuffield Department of Clinical Neurology, University of Oxford, Oxford OX3 9DU, UK

**Keywords:** Oscillations, Human, Intracranial recordings

## Abstract

Functional neurosurgical techniques provide a unique opportunity to explore patterns of interaction between the cerebral cortex and basal ganglia in patients with Parkinson's disease (PD). Previous work using simultaneous magnetoencephalographic (MEG) and local field potential (LFP) recordings from the region of the subthalamic nucleus (STNr) has characterised resting patterns of connectivity in the alpha and beta frequency bands and their modulation by dopaminergic medication. Recently we have also characterised the effect of movement on patterns of gamma band coherence between the STNr and cortical sites. Here we specifically investigate how the prominent coherence between the STNr and temporal cortex in the alpha band is modulated by movement both on and off dopaminergic medication in patients following the insertion of Deep Brain Stimulation (DBS) electrodes. We show that movement is associated with a suppression of local alpha power in the temporal cortex and STNr that begins about 2 s prior to a self-paced movement and is independent of dopaminergic status. In contrast, the peak reduction in coherence between these sites occurs after movement onset and is more marked in the on than in the off dopaminergic medication state. The difference in alpha band coherence on and off medication was found to correlate with the drug related improvement in clinical parameters. Overall, the movement-related behaviour of activities in the alpha band in patients with PD serves to highlight the role of dopamine in modulating large-scale, interregional synchronisation.

## Highlights

► We studied subthalamo-cortical coherence in the alpha band in Parkinson's patients. ► A decrease in coherence occurred with movement facilitated by dopamine. ► This effect correlated with clinical improvement.

## Introduction

It is now widely believed that exaggerated beta band (13–30 Hz) activity in the cortico-basal ganglia circuit is an important pathophysiological abnormality in Parkinson's disease contributing particularly to bradykinesia (Eusebio et al., 2009; Hammond et al., 2007; Uhlhaas and Singer, 2006; Weinberger et al., 2009). However, important interactions occur in other frequency bands, and much less is known about how these may contribute to both normal movement and to the pathophysiology of Parkinson's disease.

Advances in neurophysiological methodology have facilitated the simultaneous recording of magnetoencephalographic (MEG) activity from the cortical surface and local field potential activity (LFP) from the basal ganglia, thus enabling the characterisation of resting patterns of connectivity between the two levels and the investigation of how this may be modulated by movement and the dopamine prodrug, levodopa (Hirschmann et al., 2011; Litvak et al., 2010, 2011a, 2012). This approach affords valuable insights into the complex oscillatory patterns of neuronal activity seen in Parkinson's disease.

Previous work has demonstrated the existence of two spatially and spectrally distinct networks at rest in Parkinsonian patients (Hirschmann et al., 2011; Litvak et al., 2011a, 2012). A temporoparietal–brainstem network was coherent with the region of the subthalamic nucleus (STNr) in the alpha (7–13 Hz) band, whilst a predominantly frontal network was coherent in the beta (15–35 Hz) band. It has been hypothesised that the beta network determines motor state whilst the alpha network may play a role in attentional processing (Hirschmann et al., 2011; Litvak et al., 2011a).

The modulation of beta band cortico-STN connectivity by movement and levodopa has previously been demonstrated in both Parkinsonian humans and in rodent models of Parkinsonism. These studies would suggest that the beta coherence between the STNr and cortex drops before and during movement and during imagination or observation of movement (Alegre et al., 2010; Cassidy et al., 2002; Kühn et al., 2006; Lalo et al., 2008; Sharott et al., 2005). Recently, using simultaneous recordings of MEG and LFP in the STNr we have also shown that the coherence in the gamma frequency (60–90 Hz) band between STNr and primary motor cortex increases with both movement and levodopa therapy. We also provided evidence that gamma band coherence is likely to be modulatory rather than related to movement processing, by showing that there were no gamma coherence differences between synchronous and sequential finger movements. Importantly the change in coherence with medication was found to correlate with the degree of improvement in bradykinesia-rigidity scores (Litvak et al., 2012). In this study of the same patients we specifically aim to characterise how the coherence between the STNr and temporal cortex in the alpha frequency band is modulated by movement and by administration of levodopa. Furthermore, we aim to determine whether coherence changes are primarily modulatory or associated with movement processing by studying sequential and synchronous finger movements. Coherence changes of similar amplitude and duration at movement outset may be expected with modulatory processes such as arousal, whilst sustained differences may be predicted during more prolonged sequential movements in the case of coherence influencing motor processing. A final key aim was to determine whether dopamine induced modulation of alpha coherence was associated with clinical change.

## Methods

### Patient and surgery

This study continues the analysis reported by Litvak et al. (2012) and is based on the same patient cohort. Seventeen patients (age 55 ± 7 years, 6 female, 2 left-handed) were studied. In one patient the surgery was performed only on the right side. All patients were diagnosed with PD according the Queen Square Brain bank criteria (Gibb and Lees, 1988). A thorough description of the DBS electrodes, surgical implantation and externalization techniques, post-operative imaging and clinical details can be found in Litvak et al. (2012). All experimental procedures had received prior approval from the local research ethics committee. The mean UPDRS part III score off medication was 47.6 (standard error 3.8) whilst the mean score on medication was 13.7 (standard error 1.5).

The patients were studied in the interval between DBS electrode implantation and subsequent connection to a subcutaneous stimulator between 2 and 7 days postoperatively.

### Experimental paradigm

The experiment was divided into blocks — several minutes of recording. The patients could rest between the blocks. Each block comprised either rest or a movement task. During rest blocks the subjects were instructed to remain still with their eyes open for 3 min (Litvak et al., 2011a). During movement blocks the subjects performed either simultaneous button presses with index, middle and ring fingers (SYN) or a sequential button presses with index, ring and then middle finger (SEQ), with either left or right hand (one kind of movement with the same hand within a block). The movements were self-paced. The subjects were instructed to move when they wanted, but not to do it too frequently and take about 15 s between movements without counting silently (see supplementary Fig. 1). Feedback to the subjects was presented visually, using Matlab (The MathWorks, Inc., Natick, MA) and a custom script based on Cogent (http://www.vislab.ucl.ac.uk/cogent.php). This script monitored the movement times and displayed messages on the screen when the inter-movement interval was shorter than 12 s (‘slow down’) or a movement sequence in the complex condition was incomplete (‘sequence incomplete’). These trials were not analysed. When performing correctly the subjects did not get any feedback and the screen just showed a fixation cross. In case of incorrect performance, the script waited to collect another movement so that it ran until 8 correctly performed movements were collected or at most for a total of 7 min. The subjects could usually complete a movement block in 3–4 min. A neurologist was present in the magnetically shielded room during the experiments to monitor the patients and their performance of the task. Up to 18 blocks were performed in each experiment. The order of the conditions was pseudo-randomised separately for blocks 1–9 and blocks 10–18; so that each half would contain one rest block and two movement blocks of each type. In all experiments at least 9 blocks were recorded, but only two patients managed to complete all 18 blocks. A single recording session lasted about 1.5–2.5 h (including preparation).

The whole experiment was performed twice: after overnight withdrawal of dopaminergic medication (OFF drug) and after the patients took at least 200 mg of levodopa (ON drug). The order of these drug conditions was counter-balanced over patients. Twelve patients were able to complete both experiments, three were only recorded ON drug and two patients could not tolerate levodopa and were, therefore, only recorded OFF drug.

The two movement patterns differed in several regards, even though the same fingers were used to enact the same key presses. In the SYN task all three fingers simultaneously depressed three keys, whereas in the SEQ task only one finger at a time was used to depress one key. Consequently the conditions differed in their initial parameterization. Nevertheless, the same three keys were eventually depressed in the SEQ task, which therefore went on for longer than synchronous button presses. Hence, similar spectral changes across the two movement types would suggest modulatory processes that were not responsible for specific elements of motor processing. Conversely, differences in oscillatory activities between movement types would suggest that spectral features may be involved in the specification of motor parameters like force or movement duration.

### LFP-MEG recordings

MEG recordings were obtained with a 275 channel system (CTF/VSM MedTech, Vancouver, Canada). Simultaneously, LFP, electro-oculographic (EOG) and electromyographic (EMG) signals were recorded using an integrated EEG system (amplification × 1, dynamic range ± 125 mV) and high-pass filtered (in hardware) above 1 Hz to avoid saturation of the amplifiers due to DC offsets. All signals were low-pass filtered in hardware below 300Hz. The data were sampled at 2400 Hz and stored to disk. Four intracranial LFP channels were recorded from each contact, on each side, and referenced to a cephalic reference. LFP recordings were converted off-line to a bipolar montage between adjacent contacts (3 bipolar channels per hemisphere; 01, 12 and 23) to limit the effects of volume conduction from distant sources. EMG data were recorded from right and left first dorsal interosseous muscles with a reference at the muscle tendon. Button presses were also recorded in all subjects.

Head location was monitored using three head position indicator (HPI) coils attached to the subject's nasion and pre-auricular points. For all but the first subject we used continuous head localization and recorded the head locations throughout the experiment. Loss of head tracking occurred intermittently in some patients, possibly due to metal artefacts disrupting the head tracking function of the MEG sensors. During offline processing we compared the instantaneous distances between HPI coils with the distances based on robust average (Holland and Welsch, 1977) of locations across the whole continuous recording. Time frames where discrepancies were detected were discarded and replaced with linear interpolation based on the other time frames. This method works well when the tracking is valid for more than half of the recording, which was the case for all recordings reported here.

### Data analysis

#### Characterisation of individual subject cortical–STNr coherence

The data were analyzed using custom Matlab scripts based on SPM8 (Litvak et al., 2011b) and Fieldtrip (Oostenveld et al., 2011) toolboxes (the Fieldtrip code we used is included in the SPM8 distribution).

Selection of STNr-LFP channels for the present study was based on the analysis reported in Litvak et al. (2011a). Briefly, for that analysis we computed sensor level coherence between each STNr-LFP channel and each MEG channel for each subject during rest over a range of frequencies generating multiple sensor level coherence images. The images for each STNr-LFP channel were stacked to produce a 3D image (with two spatial and one frequency dimensions), which was subsequently subjected to statistical comparison with trial-shifted data in SPM8 in order to determine frequency ranges in which there was statistically significant coherence between the STNr-LFP channel and MEG channels at rest. For the purposes of the present analysis we selected only those STNr contact pairs for each subject with statistically significant coherence with MEG channels in the alpha frequency band (see Table 1 for the chosen channels). Importantly for some subjects, different STNr channels had significant resting alpha coherence ON and OFF medication. To ensure balanced channel selection for comparing the ON and OFF conditions we selected all channels that were significant in either the ON or OFF condition or significant in both conditions and included them all in subsequent analyses for each medication condition. In this way ON and OFF analyses were of matched STNr contact pairs.

Cortical sources coherent with the selected STNr-LFP channels in the alpha (7–13Hz) frequency band were localised using the Dynamic Imaging of Coherent Sources (DICS) beamformer (Gross et al., 2001). The source localisation was performed anew for the present study using data from the movement rather than resting (as in Litvak et al., 2011a) blocks. Given that movements were performed approximately at 12 s intervals we used a temporal window of 8 to 5 s prior to movement across all trials for the purpose of DICS beamformer source localisation. We believed this would provide a good approximation to the resting state since activity in this time window is unlikely to be influenced by the preparation, execution or reafference of motor processes. Trials where the LFP data were contaminated by artefacts were rejected prior to DICS analysis (see below for rejection criteria). In subsidiary analysis we also performed beamforming in the time window between 0.5 s before movement to 1.5 s after movement. The purpose of this was to determine if there were any STN–cortical networks in the alpha band that are quiescent at rest and activated by movement.

Lead fields were computed using a single-shell head model (Nolte et al., 2004) based on an inner skull mesh derived by inverse-normalizing a canonical mesh to the subject's individual preoperative MRI image (Mattout et al., 2007). Coregistration between the MRI and magnetoencephelography coordinate systems used three fiducial points: nasion, left and right pre-auricular (see Litvak et al., 2010 for further details). The coherence values were computed on a 3D grid in Montreal Neurological Institute (MNI) space with spacing of 10 mm bounded by the inner skull surface. Values at the grid points were then linearly interpolated to produce volumetric images with 2 mm resolution. These images were smoothed with a 5 mm isotropic Gaussian kernel.

#### Identification of group and individual coherence peaks

To identify the cortical areas consistently coherent with the STNr across the group we computed the average of all the individual images that had been generated in the 8 s to 5 s pre-movement period. Importantly, these images had been normalised prior to averaging, by dividing the coherence at each beamformer grid point by the mean of that image. This ensured that each included hemisphere-contact pair contributed equally to the calculation of the average. All images corresponding to left STNr contacts were flipped across the mid-sagittal plane to allow comparison of ipsilateral and contralateral sources to the STN regardless of original STN side. The global maximum of the resulting image was defined as the cortical source maximally coherent with the STNr across all subjects.

Subsequently, for each subject and each contact we selected from the individual image, the peak closest to this group peak. The individual cortical peaks were then used for time series extraction. This approach represented a compromise between a systematic choice of site and maximisation of coherence. Crucially, in order to ensure immunity from artefact the orientation of the cortical source was defined as the normalised imaginary part of the cross-spectral density vector between the STN-LFP and the three orientations of the cortical source (Litvak et al., 2010; Nolte et al., 2004).

#### Source activity extraction

Source activity extraction was performed using the Linearly Constrained Maximum Variance (LCMV) beamformer (Van Veen et al., 1997) with DICS beamformer-identified sources and time epochs from − 8 to 5 s relative to the button press. Beamformer filters were computed for each trial separately with a trial-specific forward model based on continuous head tracking data. This was done to reduce the confounding effects of head movements and varying numbers of trials across subjects and conditions. We refer the reader to Litvak et al. (2012) for a more detailed discussion of this issue. We used a beamformer regularization of 0.01% of the signal variance (averaged over channels) as this value was shown to be optimal in our previous studies (Litvak et al., 2010).

#### Preprocessing of virtual electrode and LFP data

The virtual electrode channels derived from the MEG sometimes contained discontinuous jumps, whose origin could be traced to occasional resets of the SQUID sensor circuitry. These jumps were detected by thresholding the differences between adjacent samples. When a jump was detected the values from 20 samples before to 20 samples after the jump were replaced by the median difference over this segment and the modified difference time series were summed again to produce the original time series with the jump corrected. The corrected data were digitally filtered (1 Hz 5th order high pass, and 4th order notch filters for 50 Hz and all harmonics up to 550 Hz, zero-phase Butterworth in all cases). Finally, prior to spectral analysis, the channel data were standardised by subtracting the mean and dividing by the standard deviation for each channel and trial separately. This ensured that all trials contributed equally to estimates of source activity.

#### Artefact suppression

In our dataset LFP, EMG and EOG data were contaminated by occasional brief electrical discharges due to a grounding problem that could not be completely resolved at the hardware level. Since the occurrence of these spikes was increased around the button press periods it is important to rule out wide-band artefacts as possible reasons for changes in low frequency amplitude or power. The preprocessed LFP and virtual electrode data series were examined for the presence of outliers by thresholding. The thresholds were set such that they could separate the artefacts from the remaining data. These thresholds were 5 standard deviations for the original data and 2 standard deviations for the beamformed source derivative.

This artefact suppression technique was employed at two stages of the present analysis. Firstly prior to DICS beamforming in the period from 8 to 5 s before movement or from 0.5 s before movement to 1.5 s after movement we removed artefact from the LFP channels in order to facilitate accurate cross spectral density estimates for beamforming. Secondly in order to preclude contribution of spikes to the button press response, trials with artefacts in the period from 0.2 before movement to 2 s after movement were excluded from the analysis of event related power. The effects of the remaining spikes were suppressed by robust averaging (see Litvak et al., 2012). Trial exclusion was performed separately for beamformed sources and STN channels to minimise data loss. For the analysis of event-related coherence we used all the trials. This was motivated by simulations showing that high-frequency artefacts in only one of the channels had a minor effect on coherence estimates — and were further suppressed by our robust procedure for coherence computation. A more detailed description of the benefits of robust averaging in this particular dataset with artefacts and relatively few trials can be found in Litvak et al. (2012). Essentially the procedure involves computing the distribution of values over trials and down weighting outliers when computing the average. This makes it possible to suppress artefacts restricted to narrow time and frequency ranges without rejecting whole trials.

#### Excluding data with high frequencies in the evoked response

To further ensure that the phenomena we report are not caused by brief artefacts we computed the averages of both the movement-related LFP and virtual electrode data in the time domain and performed time-frequency analysis of these evoked responses using the same settings as for single trials. Even after exclusion of trials containing artefacts, for some hemispheres we found high-frequency activity around the button press. All of these cases were from the beamformed sources, and not from STNr-LFPs. Not all of this activity was clearly artefactual, but since this was a likely explanation we excluded all contact-hemisphere pairs where such activity was found from our analysis of beamformed source power. 15 contact-hemisphere pairs were removed ON medication (from 9 different subjects) and 4 hemisphere contact pairs were excluded OFF medication (for 3 different subjects. Table 1 details the data included in the analysis.

### Spectral analysis

For efficient spectral estimation from a relatively small number of trials we used multitaper spectral analysis (Thomson, 1982). This method is based on premultiplying the data with a series of tapers optimised for producing uncorrelated estimates of the spectrum in a given frequency band. This sacrifices some of the frequency resolution, in a controlled manner, to increase signal to noise ratio. It does this by effectively multiplying the number of trials by the number of tapers used. We estimated the spectra between − 8 and 5 s relative to the first button press of each trial, in overlapping windows of 400 ms (shifted by 50 ms). The frequency resolution was set to the inverse of the time window (2.5 Hz) for up to 25 Hz, then 0.1 times the frequency for 25 to 50 Hz and then to a constant 5 Hz resolution. These settings resulted in a single taper being used for 2.5–30 Hz, 2 tapers for 32.5–42.5 Hz and 3 tapers for 45 Hz and above. The resulting time–frequency images had no discontinuities thanks to the continuous frequency resolution function. The time–frequency images were then averaged using robust averaging (Holland and Welsch, 1977; Wager et al., 2005; Litvak et al., 2012) and percentage change time–frequency responses were obtained by normalizing to the baseline (8 to 5 s) before button press.

For the purposes of statistical analysis, given our focus on the alpha frequency band (7–13 Hz), we averaged the spectral data over this band by computing a multitaper spectral estimate with central frequency of 10 Hz and frequency resolution of 3 Hz. This is a more efficient way of spectral estimation than averaging over pre-computed time-frequency images with higher frequency resolution (Mitra and Pesaran, 1999). The power time series were then averaged using the robust averaging procedure described above.

Coherence was estimated using a similar spectral estimation procedure except that robust averaging was used during coherence computation and was applied separately to the absolute values of the cross-spectral density and to the power of the two sources (Litvak et al., 2012). In the case of cross-spectra, the weights computed from the absolute values were then applied to the complex cross-spectra when computing the mean coherency over trials. Percent changes in cross-spectral responses were computed as above.

### Statistical analysis of time–frequency data

Statistical analysis was performed using Statistical Parametric Mapping (as implemented in SPM8). This treats the time series of alpha power or coherence as images with up to 3 dimensions allowing the identification of time windows showing significant effects over subjects, while controlling for the multiple comparisons using random field theory (Kilner et al., 2005; Litvak et al., 2011b).

The results of time–frequency analysis were exported to Neuroimaging Informatics Technology Initiative (NIfTI) format and smoothed with a Gaussian smoothing kernel with a Full Width Half Maximum (FWHM) of 500 ms. All the reported findings are significant with family-wise error (FWE) correction (p < 0.05). In the results we also report the peak t statistic with the corresponding p values.

To test for the effects of experimental conditions we constructed a 2 × 2 × 2 repeated measures Analysis of Variance (ANOVA) with factors ‘Task’ (SYN vs. SEQ), ‘Drug’ (ON vs. OFF) and ‘Side’ (Ipsilateral vs. Contralateral to the selected STN channel). In this design we included subject and side covariates in order to account for resulting dependencies in the error. Significant features of the mean responses were determined by subjecting mean images across conditions for power and coherence to a single-sample t-test across subjects.

To test for correlation with clinical scores we performed an Analysis of Covariance (ANCOVA) by adding to the ANOVA described above a regressor with contralateral hemibody bradykinesia-rigidity scores. The scores comprised the sum of items 22–26 of the UPDRS part III score. Motor examination was performed pre-operatively, after withdrawal from medication overnight, in a practically defined OFF state (so that patients had their last antiparkinsonian medication 9–12 h prior to testing). Motor examination was repeated on the same day 1 h after their usual antiparkinsonian treatment, provided the levodopa dose was at least 200 mg. Where this was not the case the patient's standard antiparkinsonian medication was replaced by a single dose of levodopa 200 mg. Tremor scores were also independently tested as clinical regressors. As mentioned, the UPDRS scores used for clinical correlations were determined pre-operatively. However, this would serve to weaken any correlation between variables. Thus any correlation between the difference in UPDRS on and off medication and the difference in coherence induced by medication may be an under-estimate.

To test for a main effect of contact pair across subjects we constructed a one way ANOVA with 3 levels, each representing the selected contact pair: 01, 12, 23. In this design we included subject and side covariates in order to account for resulting dependencies in the error.

### Additional statistical analyses

To show that the alpha network during movement had the same topography as during rest we performed a paired t-test of the 3D beamformer images comparing the period from 0.5 s before movement to 1.5 s after movement for all subjects and conditions.

To show that the MNI co-ordinates ON and OFF medication were not significantly different to each other we used the Hotelling's t-squared statistic and its chi-squared approximation.

For the behavioural data, we pooled the timings of all button presses after the first press for all subjects separately for the complex and simple tasks on and off medication. The mean timing was then computed for each of the four conditions for each subject. Subsequently we ran a repeated measures 2 × 2 ANOVA with factors ‘Drug’ (ON vs. OFF) and ‘Task’ (SYN vs. SEQ).

## Results

### Topography of STN–cortical alpha coherence during movement and pre-movement

Paired t-test did not reveal any cortical areas where coherence with the STN was greater in the movement period (0.5 s before movement to 1.5 s after movement) than in the premovement period (8 s to 5 s prior to movement). See supplementary Fig. 3 for further details.

### Cortical source localisation

**Fig. 1:**
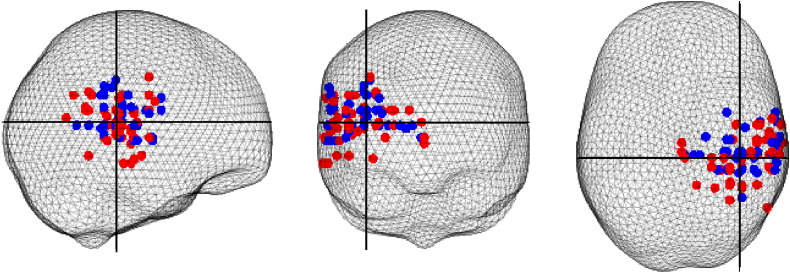
Cortical alpha peaks of coherence closest to the group peak (shown by the intersection of the black cross hairs) are plotted for all subjects and contacts both ON and OFF dopaminergic medication (ON, shown in red and OFF shown in blue).

Cortical source localisation of alpha activity coherent with the STNr in the interval of 8 s to 5 s before movement was performed for the 52 STNr contact pairs demonstrating coherence with the temporal cortex in the 17 subjects ON and OFF medication (Fig. 1). The location of the group peak in MNI co-ordinates was 38 − 32 12, corresponding to the right superior temporal gyrus. The mean distance of the selected peaks from the group peak in the MNI space was 22.2 mm (standard error 1.05 mm). Furthermore, the mean MNI co-ordinates ON and OFF medication were 42 31 13 and 42 29 16 respectively. The Hotelling's t-squared test did not support the hypothesis that the coordinates of sources localised ON medication were significantly different to those localised OFF medication (χ^2^ = 3.6, df = 3, p = 0.31). Individual subject source locations in MNI coordinates and the selected contacts for DICS beamforming are detailed in Table 1.

### Responses in cortical sites and STNr induced by voluntary movement

**Fig. 2:**
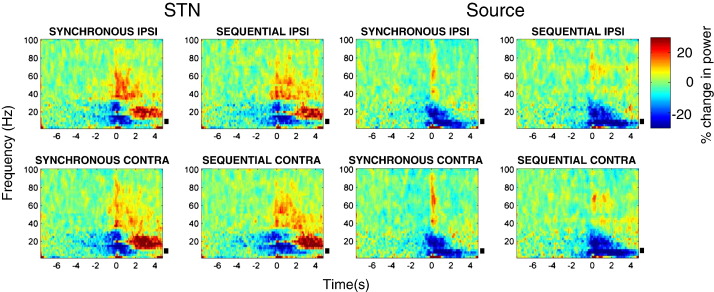
Time–frequency images of power, averaged across subjects, for each of the four conditions OFF dopaminergic medication. We used a baseline period from − 8 to − 5 s prior to movement with power changes reported as percentage change. There is a beta desynchronisation with onset prior to movement and gamma power increase upon movement. The black bars indicate alpha band frequencies between 7 and 13 Hz.

**Fig. 3:**
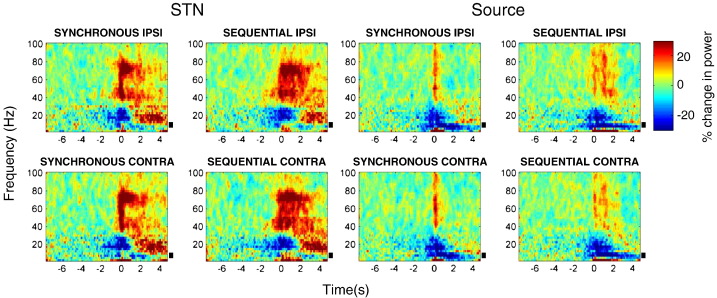
Time–frequency images of power, averaged across subjects, for each of the four conditions ON dopaminergic medication. We used a baseline period from -8 to − 5 s prior to movement with power changes reported as percentage change from this. Gamma power increases are more marked than OFF medication (Fig 2). The black bars indicate alpha band frequencies between 7 and 13 Hz.

**Fig. 4:**
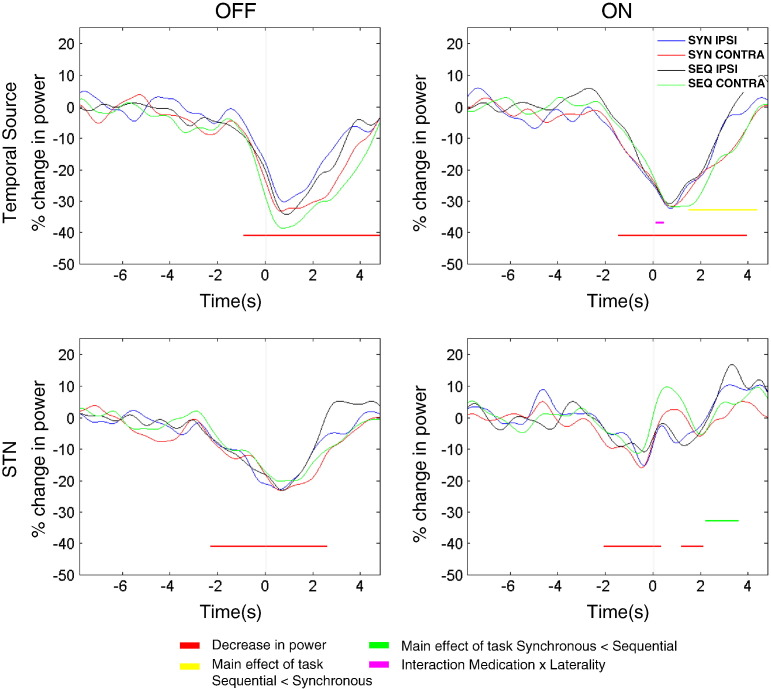
Time series of alpha (7–13Hz) power for all four conditions averaged across subjects for the source and STNr both ON and OFF dopaminergic medication. The time series are baseline-corrected from − 8 to − 5 s prior to movement and the changes are shown as percentage changes from baseline. Time series have been smoothed by a 500 ms FWHM Gaussian kernel. The power changes were subjected to statistical analysis, separately for the STNr and source.

One way ANOVA, revealed no significant effect of the selected contact pair for power spectra from the STNr or cortical sites. The peak F statistic for cortical contact pair power was F_(**2**,**287**)_ **= 2**.**8**, with a corresponding p value > 0.05). Similarly for the STN, the results were: F_(**2**,**287**)_ **= 2**.**4**, p > 0.05. Figs. 2 and 3 show the power changes induced by button presses at both the beamformer extracted source locations and the STNr channels, averaged across all subjects and hemispheres for each of the four different experimental conditions ON and OFF medication. In the STNr we see a characteristic event related desynchronisation (ERD) in beta power prior to and during movement, followed by a beta event related synchronisation (ERS). There is also a reduction in alpha activity both prior to and during movement and a broadband gamma ERS associated with movement onset. Activity in the extracted cortical sources is dominated by a beta and alpha power decrease upon movement. Since we were explicitly interested in modulations of alpha band power, these time series are plotted for both the STNr and cortical sources in Fig. 4.

In the cortical sources there was a significant ERD in alpha power occurring prior to movement and continuing up to 4 s after movement, both ON and OFF dopaminergic medication (shown by the red lines in Fig. 4). The peak t-scores and their corresponding p values are as follows: OFF t_282_ = 4.2, p < 0.01 and ON t_282_ = 5.7, p < 0.01) For sake of consistency we display the main effects and interactions in Figs. 4 and 6 in the ON plots only. There was also a significant main effect of movement task with sequential movements producing a greater and more prolonged alpha desynchronisation between 1.5 s and 4.3 s after movement onset (shown by the yellow bar in Fig. 4 — peak t_282_ = 3.3, p < 0.05; also see supplementary Fig. 5). The latter may reflect the longer duration of the sequential movements. In addition to this, there was a narrow period of significant interaction between medication and the laterality of movement, with contralateral movements producing greater alpha desynchronisation than ipsilateral movements predominantly OFF medication (shown by the pink bar in Fig. 4 — peak t_282_ score 3.4, p < 0.05).

For the STNr contacts, we observed a significant reduction in alpha power from about 2 s before movement in both the ON and OFF medicated states. OFF medication the peak t_282_ score was 4.6 corresponding to a p value of < 0.01. Similarly ON medication the peak t_282_ score was 3.5, with a corresponding p value of < 0.05. There was significant but delayed main effect of task, shown by the green bar in Fig. 4, which was opposite in direction to that observed for temporal sources (peak t_282_ = 3.4, p < 0.05; Also see supplementary Fig. 5). This might reflect a more delayed event-related synchronization following movement in the sequential task. Thus the main effect of task at both cortical and STNr levels might reflect the different durations of the tasks, although whether this was more manifest in the timing of the power suppression or power rebound was dependent on level.

### Reactivity patterns of STN–alpha source coherence

**Fig. 5:**
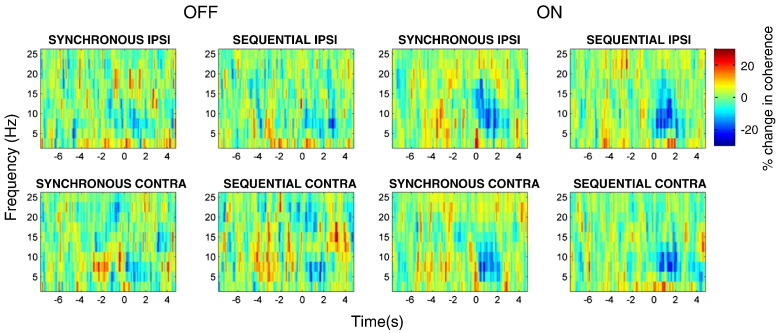
Time–frequency images of coherence, averaged across subjects, for each of the four conditions OFF and ON dopaminergic medication. The coherence spectra have been smoothed by a 2D Gaussian kernel (FWHM of 2.5Hz and 500 ms). We used a baseline period from − 8 to − 5 s prior to movement with coherence changes reported as percentage change from this. Movement related changes dominate in the alpha band.

**Fig. 6:**
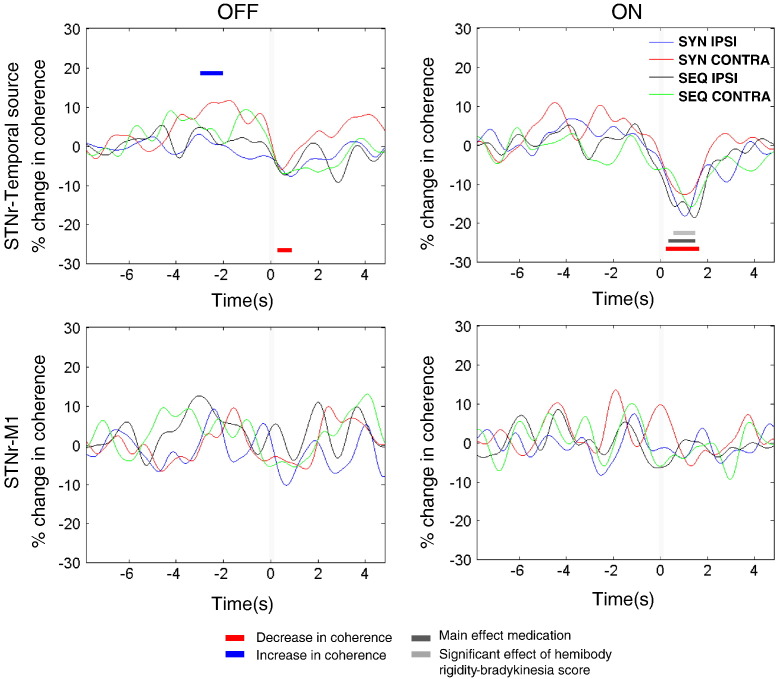
Time series of alpha (7–13Hz) cortical source–STNr coherence averaged across subjects, separately for ON and OFF medication. Changes are shown relative to the baseline from − 8 to − 5 s prior to movement for all conditions and subjects. Time series have been smoothed by a 500 ms FWHM Gaussian kernel. Upper panel pair: Temporal–STNr coherence. Note that the significant main effect of medication, given by the dark grey line, was due to coherence being greater OFF than ON medication. Lower panel pair: M1–STNr coherence, where there are no significant changes.

One way ANOVA, revealed no significant effect of the selected contact pair for coherence spectra (peak F_(**2**,**287**)_ **=** 1.9, p > 0.05). Fig. 5 displays the changes in coherence induced by button presses between the beamformer extracted source locations and the STNr channels, averaged across all subjects and hemispheres for each of the 4 different experimental conditions ON and OFF medication. Coherence is displayed at frequencies below 25 Hz as statistically significant movement related changes were not seen at higher frequencies (see supplementary Fig. 4). Note that the majority of the change in coherence occurs in the alpha rather than beta frequency bands. The time series of the alpha band coherence between STNr and temporal sources is given in the upper panels of Fig. 6. Both ON and OFF medication there was a significant drop in coherence compared to baseline upon movement (red bars in Fig. 6). The peak t scores and the corresponding p values for these effects were as follows: OFF t_282_ = 4.1, p < 0.01 and ON t_282_ = 5.8, p < 0.01. There was an additional main effect of medication in which coherence was significantly less ON than OFF medication after movement (dark grey bar in Fig. 6, peak t_282_ = 4.8, p < 0.01; Also see supplementary Fig. 5). Finally, OFF medication there was a narrow time window during which alpha coherence rose prior to movement (peak t_282_ = 3.2 p < 0.05). For reference, the lower panel in Fig. 6 gives the time series of the alpha band coherence between STNr and the primary motor cortex (M1) source (as defined in Litvak et al., 2012). This makes it clear that alpha coherence changes are specific to the temporal cortex. Indeed no significant changes were found when STNr-M1 alpha band coherence was subjected to the same ANOVA as STN-temporal alpha band coherence.

### Correlation with clinical variables

**Fig. 7:**
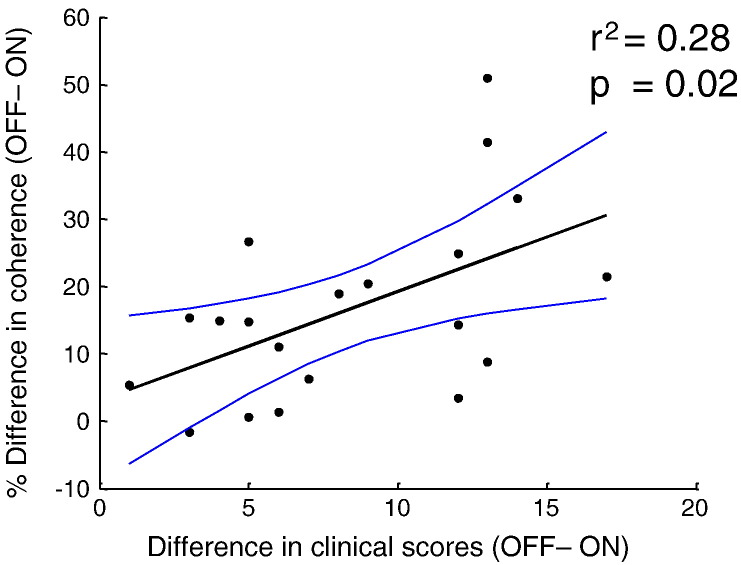
Scatter plot of data for 20 hemispheres in 11 subjects (one with only unilateral electrode implantation and one with unilateral STNr channel selection based on the selection criteria discussed in the Methods) with paired recordings ON and OFF medication. Difference in hemibody rigidity and bradykinesia scores ON and OFF medication have been plotted against the difference in alpha coherence ON and OFF medication in the interval from 0.5 to 1.45 s after movement onset (averaged across contact pairs, where more than one was coherent with temporal cortical activity). This was the interval over which there was a significant correlation with the hemibody rigidity and bradykinesia score. The r squared value and the corresponding p value of the linear regression (F = 7.14, df = 1,18) are displayed in addition to confidence intervals for the line of best fit, shown in blue.

To assess the possible clinical relevance of the spectral changes described above, we performed an ANCOVA, with the contralateral hemibody bradykinesia-rigidity scores as regressor. No significant correlations were found with either cortical source or STNr alpha power. However, significant clinical correlations with STNr—cortical source alpha coherence were identified following the initial button press, whereby bigger movement related drops in alpha coherence with levodopa were associated with bigger improvements in clinical state with levodopa. These were within the time period for which there was a main effect of drug (light grey bar in Fig. 6). To demonstrate that the correlations with cortical–STNr alpha coherence were not driven by outliers we plotted the relations between these effects and clinical improvement for individual hemispheres (Fig. 7). We also separately correlated hemi-body tremor scores with alpha power and coherence, but found no significant effects. Finally, we considered the question whether the STNr–M1 gamma coherence and STNr gamma power previously shown to correlate with the same clinical scores explain the same part of the variance as the STNr–cortical alpha coherence. Accordingly, we included all three factors in a multiple regression model. Although the model was significant (F_(3,16)_ = 3.68, p = 0.03, r^2^ = 0.41), none of the three factors made a significant independent contribution.

### Behavioural data

**Fig. 8:**
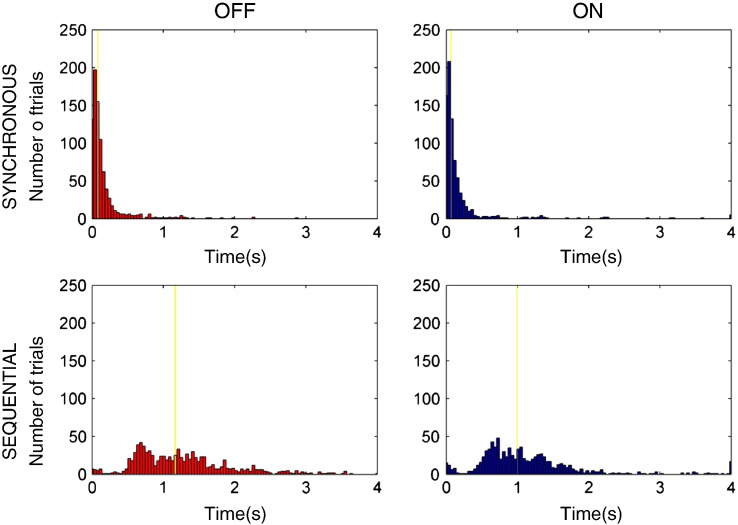
Histograms of all button press timings across all subjects, for the sequential and synchronous tasks on and off medication. The initial button press was at 0 s. The yellow lines indicate the median timings. The numbers of button presses shown for the four conditions are as follows: (1) synchronous on — 792 (2) synchronous off — 830 (3) sequential on — 917 (4) sequential off — 934.

In Fig. 8 we plot for all trials of all subjects the distributions of the button press timings after the first press (which occurred at time 0 s) for the complex and simple tasks on and off medication. The median timings are indicated by the yellow lines. There was a significant main effect of task type with the sequential task resulting in longer intervals between the first and all subsequent button presses (F(1,13) = 7.8, p = 0.01). Furthermore there was a significant main effect of medication (F(1,13) = 5.3, p = 0.04) and also a significant interaction between task type and medication (F(1,13) = 4.9, p 0.04) suggesting that the effect of dopamine on reducing timing intervals was greater in the sequential rather than the synchronous task.

## Discussion

Previous work using simultaneous MEG and LFP recordings highlighted the existence of a resting alpha band network between the STNr and temporal cortical areas which was not modulated by dopaminergic state in patients with Parkinson's disease (Hirschmann et al., 2011; Litvak et al., 2011a). Here we show that the alpha frequency band coherence between temporal cortical areas and the STNr is reduced following movement onset and that the degree of suppression in alpha coherence is significantly greater ON than OFF dopaminergic medication. In contrast, alpha power was suppressed up to two seconds before movement and this was unaffected by dopaminergic medication.

The present findings are in several ways the reciprocal of the gamma band coherence between STNr and M1 reported in the same patients (Litvak et al., 2012). Alpha band STNr-temporal cortex coherence was suppressed with movement, particularly after dopaminergic medication. Conversely gamma band STNr–M1 coherence was increased with movement, particularly after dopaminergic medication. Moreover, the two correlated with clinical state but with opposite polarity. These two correlations, however, were not independent, explaining the same portion of the variance in the change in clinical scores upon treatment. For both, correlations were limited to bradykinesia and rigidity in the contralateral limb. Furthermore, both alpha and gamma coherences were unaffected by the type of movement performed. In other words the increase in gamma and the decrease in alpha were little different in terms of size or duration between sequential or synchronous finger movements. The behavioural data in contrast displayed significant differences in the button press timings between the two movement types. Furthermore, in keeping with dopamine induced improvements in clinical state, we found that the time intervals between the first and successive button presses were reduced in the on medicated state. The above observations lead to the tentative suggestion that the two coupled systems are functionally reciprocally related (see Supplementary Fig. 2). In other words, around the time of movement our PD patients may have to disengage the STNr from its locking to temporal cortex, which is preferentially at alpha band frequencies, and engage STNr locking to M1 in the gamma band. Dopamine appears necessary for this task-related switch in coupling. Granger causality analysis suggests that coherence is predominantly driven by the temporal lobe in the alpha band (Litvak et al., 2011a) and by the STNr in the gamma band (Litvak et al., 2012). The functionality of this relationship, as suggested by a lack of effect of task type, is likely to be modulatory to movement and related to arousal or attention rather than directly to motor processing.

The above hypothesis, although speculative, is consistent with the effects of direct stimulation. Stimulation of the STNr at 10 Hz has been reported to increase bradykinesia (Timmermann et al., 2004), whereas stimulation of the STNr and M1 at gamma band (60–90 Hz) frequencies facilitates movement (Joundi et al., 2012; Tsang et al., 2012). Treatment with levodopa, which facilitates the above switch in STNr connectivity, also improves movement.

The task-related decrease in coupling with temporal cortex identified here is reminiscent of the task-related deactivation of the default mode attention network (Raichle and Snyder, 2007). The parallels are heightened by the association of the latter network with alpha band synchronization (Jann et al., 2009; Knyazev et al., 2011), and by evidence that its task-related deactivation is facilitated by dopamine (Nagano-Saito et al., 2009). These parallels serve to highlight the general role of dopamine in modulating large-scale, interregional networks and their interactions (Dang et al., 2012; Gordon et al., 2012; Kelly et al., 2009; Walters et al., 2000). Task-related amplitude and frequency modulations in the beta band have also been demonstrated in the STN and shown to be influenced by dopaminergic state (Foffani et al., 2005).

An alternative explanation for the present findings is that alpha band coherence relates to the presence of rest tremor, to which it is harmonically related. Rest tremor most often disappears with voluntary movement, perhaps accounting for the task-related modulation of alpha coherence. However, coherence with temporal cortex was not a feature of the previously described alpha network coherent in parkinsonian rest tremor (Pollok et al., 2009; Timmermann et al., 2004). Moreover few patients in this study and in that by Hirschmann et al. (2011) had appreciable tremor. Finally, the correlation between treatment-related changes in alpha coherence and corresponding changes in clinical state was limited to bradykinesia-rigidity and not tremor.

Importantly, we found no significant effect of the selected contact pair for power or coherence spectra. Consequently we found no evidence to suggest that the movement related coherence changes observed are limited to a particular region of the STN.

Note that we have been cautious in ascribing function to precise anatomical regions. As previously discussed, there is good evidence that the LFP and MEG cannot be contaminated by one another, but whether the alpha activity in the STNr LFP originates within the STN or from a broader brainstem region is unclear (Hirschmann et al., 2011; Litvak et al., 2011a). For example, prominent alpha activity has been reported in LFPs recorded from the region of the pedunculopontine nucleus (Androulidakis et al., 2008; Thevathasan et al., 2012). In the case of MEG, we have confined ourselves to discussion of cortical regions rather than precise cortical areas, given the spatial noise introduced by metal artefacts and head movements in our patients.

We should also point out that in the present analysis we have found no evidence for the possibility of STN–cortical networks that are quiescent at rest, and become active during movement. Another feature deserving comment is the relationship between the alpha band network in the resting state and the resting beta band coherence between the STNr and frontal motor areas. The latter may also show suppression with movement (Cassidy et al., 2002; Lalo et al., 2008), as well as during imagination of movement (Kühn et al., 2006) and during action observation (Alegre et al., 2010). Although we were unable to confirm a significant suppression of beta band coherence upon movement in the current patient group (Litvak et al., 2012), we did see a negative correlation between levodopa effects on coherence in this band and corresponding changes in bradykinesia and rigidity. Thus the beta band coherence between the STNr and frontal motor areas and the alpha band coherence between STNr and temporal cortex may both represent networks that have to be disengaged during effective movement. Why there should be two such systems is a question for future work.

It is important to highlight that the processes underpinned by alpha band coherence and local regional alpha band power may be functionally distinct. This is underscored by the different temporal patterns of modulation upon self-paced movement. Thus, although alpha band coherence dropped after the onset of movement, alpha band power local to the STNr and temporal cortical source was suppressed up to two seconds before movement onset. Furthermore, there was no main effect of medication on alpha power, but there was on STNr–temporal cortex coherence. Indeed, rather than the greater movement-related drop in coherence seen with levodopa, alpha power in the cortical source contralateral to movement showed greater decreases OFF levodopa. The functional relationships of alpha power at the temporal and subthalamic levels remains unclear, although cortical activity in this frequency band has previously been related to visuospatial attentional processing, with power typically reducing in anticipation of an upcoming action (Foxe et al., 1998; Gould et al., 2011; Rohenkohl and Nobre, 2011; Thut et al., 2006).

In summary, we have demonstrated a STNr–temporal cortex alpha band network that is maximally active at rest and is disengaged with movement. Impaired disengagement of this network with movement may be a significant feature of the hypodopaminergic state in PD and appears to be ameliorated by dopaminergic therapy. An important direction for future study will be to examine the relationship between this alpha network and previously identified spatially and spectrally distinct STNr–cortical networks in the beta and gamma frequency bands which are also believed to play key roles in movement control.

## Figures and Tables

**Table 1:** Table showing the selected contacts for each subject and whether the subject was recorded ON or OFF medication, or both. In all 12 subjects had both ON and OFF recordings. In the second column of the table * indicates that the channel showed statistically significant resting alpha coherence with the cortex in the corresponding medication state (either ON or OFF medication or both). The third column indicates the MNI location of the selected peak, closest to the group peak for the corresponding channel. Channels rejected from the analysis of event related power due to the presence of high frequency artefact within the corresponding beamformed temporal sources are also listed. Note for subject 13 that the entire ON recording was excluded due to broadband artefacts within the beamformed temporal sources for all selected contacts (see also Litvak et al., 2012).

Case	Selected channel(s)	MNI co-ordinates of source(s)	Rejected channels for analysis of source power/other comments
1 OFF	L23*	− 24 − 40 10	
R12*	65 − 18 0
R23*	63 − 8 0
2 ON	L01	− 46 − 28 30	R01,R23
R01*	52 − 10 20
R23	60 − 18 12
2 OFF	L01*	− 38 − 28 20	
R01	42 − 12 20
R23*	52 − 10 18
3 ON	L12	− 10 − 50 20	L23
L23	− 22 − 52 20
R01	65 − 30 28
R12	57 − 65 15
3 OFF	L12*	− 12 − 30 8	
L23*	− 8 − 30 10
R01*	60 − 30 28
R12*	50 − 28 24
4 ON	R12	62 − 22 6	R12
4 OFF	R12*	52 − 8 2	
5 OFF	R12*	60 − 20 0	
R23*	50 − 22 6
6 ON	L01*	− 60 − 28 10	
L12*	− 56 − 20 0
L23*	− 64 − 27 − 15
R12*	58 − 15 − 10
7 ON	L01*	− 32 − 48 30	
L12*	− 32 − 60 30
L23*	− 32 − 50 32
R12*	67 − 22 15
7 OFF	L01	62 − 40 2	
L12	− 40 − 40 36
L23*	− 36 − 32 40
R12	38 − 42 30
8 ON	L01*	− 58 − 30 8	R01, R12, L12
L12*	− 58 − 38 0
L23*	− 44 − 30 6
R01*	26 − 40 2
R12	32 − 38 10
R23*	60 − 28 30
8 OFF	L01	− 48 − 32 10	
L12*	− 40 − 30 10
L23	− 28 − 28 18
R01*	30 − 40 10
R12*	38 − 30 10
R23*	32 − 40 10
9 ON	L23*	0 − 22 − 2	
R23	48 − 32 4
9 OFF	L23	− 2 − 30 2	
R23*	16 − 18 10
10 ON	L01*	− 20 − 32 20	
L12*	− 20 − 38 10
R01	30 − 30 12
R23*	40 − 32 8
10 OFF	L01	− 40 − 58 10	
L12	− 30 − 2 28
R01*	40 − 50 10
R23	30 − 28 20
11 ON	L01*	− 50 − 20 18	L01
R12	48 − 30 18
R23*	50 − 30 12
11 OFF	L01	− 62 − 2 20	L01
R12*	60 − 22 22
R23*	62 − 24 30
12 ON	L12*	− 50 − 50 10	L12
12 OFF	L12*	− 40 − 40 10	L12
13 ON	L23*	− 50 − 28 10	R12, R23, L23, R01 Entire ON recording excluded
R01*	30 − 30 20
R12*	28 − 32 20
R23*	28 − 32 20
13 OFF	L23*	− 64 − 20 10	L23, R23
R01*	38 − 30 12
R12	32 − 20 20
R23	30 − 20 20
14 ON	L12	− 66 − 10 2	L12
L23*	− 50 − 22 8
R12	36 − 10 42
R23	40 − 38 16
14 OFF	L12*	− 63 − 35 35	
L23	− 40 − 28 12
R12*	48 − 40 4
R23*	50 − 42 8
15 ON	L01	− 40 − 52 22	L01
R01*	34 − 20 − 12
15 OFF	L01*	− 48 − 40 30	
R01*	40 − 40 32
16 ON	L01*	− 40 − 50 10	
L23*	− 32 − 40 32
R01*	50 − 10 30
R23*	60 − 6 26
17 ON	L12*	− 68 − 18 − 10	

## Appendix A Supplementary data

Supplementary Fig. 1. A schematic of the motor tasks. The letters I, M and R indicate the positions of the Index, Middle and Ring fingers on the buttons of the button box. In the sequential motor task, drawn to the left of the figure, patients were instructed to move sequentially in order to depress the buttons. In the synchronised motor task, patients were instructed to move all three fingers so as to depress the buttons synchronously. The movements were self paced and performed every 12–15 s. The right and left hands were tested separately. Supplementary Fig. 2. Here we plot the time courses of coherence in: 1) the gamma band (shown in blue) between STN and beamformer identified primary motor cortex (M1), data taken from Litvak et al., 2012, and 2) the alpha band between STN and temporal cortex. The data were averaged across all subjects and conditions and smoothed with a 500 ms FWHM Gaussian kernel. The shaded areas represent standard errors of the mean. Movement is associated with a reduction in alpha band STN-temporal coherence and an increase in STN-M1 gamma band coherence. Supplementary Fig. 3. Mean across subjects of the normalised DICS images. The images have been thresholded such that only voxels with coherence greater than 2 standard deviations above the mean (1 unit) are displayed. *Yellow image*: the pattern of STN–cortical coherence in the window between 8 s and 5 s prior to movement. *Red image*: STN–cortical coherence in the window between 0.5 s before movement and 1.5 s after movement. Both images are superimposed onto a T1 weighted canonical MRI. Coronal, sagittal and axial sections through the image are displayed, oriented to the group peak (shown by the cross hairs) in the window between 8 s and 5 s prior to movement. A paired t-test (see Results) failed to reveal any cortical areas where coherence in the red image was greater than that in the yellow image. Supplementary Fig. 4. Time–frequency images of coherence (between 25 and 100 Hz), averaged across subjects, for each of the four conditions OFF and ON dopaminergic medication. As per Fig. 5, the coherence spectra have been smoothed by a 2D Gaussian kernel (FWHM of 2.5Hz and 500 ms). We used a baseline period from − 8 to − 5 s prior to movement with coherence changes reported as percentage change from this. There are no significant high frequency coherence changes. Supplementary Fig. 5. Bar plots of the main effects for power and coherence. The baseline percentage changes in power or coherence corresponding to the main effects averaged over subjects and the entire time window of significance (shown in Figs. 4 and 6) are presented with standard errors of the mean.
