# Supplementary material for: Movement related dynamics of subthalmo-cortical alpha connectivity in Parkinson's disease
Source: Neuroimage. 2013 Apr 15;70:132–42. doi: 10.1016/j.neuroimage.2012.12.041 (PMC3591253; doi:10.1016/j.neuroimage.2012.12.041)
Supplement: Supplementary file 1 — Supplementary Fig. 1. A schematic of the motor tasks. The letters I, M and R indicate the positions of the Index, Middle and Ring fingers on the buttons of the button box. In the sequential motor task, drawn to the left of the figure, patients were instructed to move sequentially in order to depress the buttons. In the synchronised motor task, patients were instructed to move all three fingers so as to depress the buttons synchronously. The movements were self paced and performed every 12–15 s. The right and left hands were tested separately. Supplementary Fig. 2. Here we plot the time courses of coherence in: 1) the gamma band (shown in blue) between STN and beamformer identified primary motor cortex (M1), data taken from Litvak et al., 2012, and 2) the alpha band between STN and temporal cortex. The data were averaged across all subjects and conditions and smoothed with a 500 ms FWHM Gaussian kernel. The shaded areas represent standard errors of the mean. Movement is associated with a reduction in alpha band STN-temporal coherence and an increase in STN-M1 gamma band coherence. Supplementary Fig. 3. Mean across subjects of the normalised DICS images. The images have been thresholded such that only voxels with coherence greater than 2 standard deviations above the mean (1 unit) are displayed. Yellow image: the pattern of STN–cortical coherence in the window between 8 s and 5 s prior to movement. Red image: STN–cortical coherence in the window between 0.5 s before movement and 1.5 s after movement. Both images are superimposed onto a T1 weighted canonical MRI. Coronal, sagittal and axial sections through the image are displayed, oriented to the group peak (shown by the cross hairs) in the window between 8 s and 5 s prior to movement. A paired t-test (see Results) failed to reveal any cortical areas where coherence in the red image was greater than that in the yellow image. Supplementary Fig. 4. Time–frequency images of coherence (between 25 and 100 Hz), averaged acr [file mmc1.pdf]

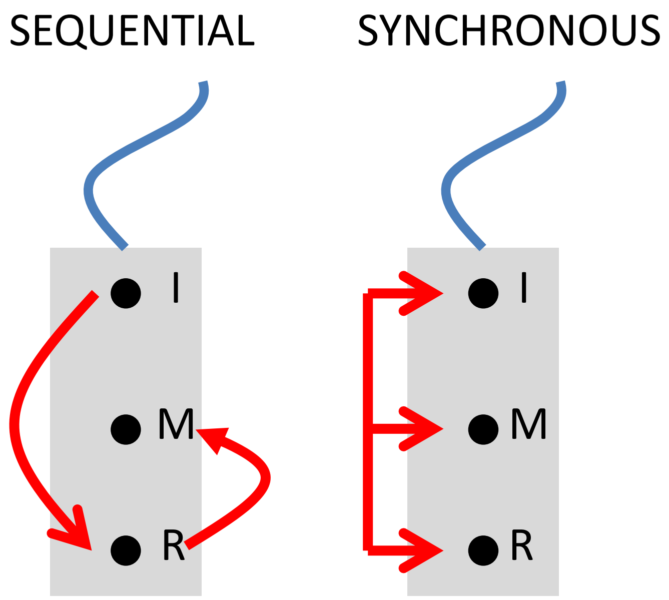

Supplementary Figure 1

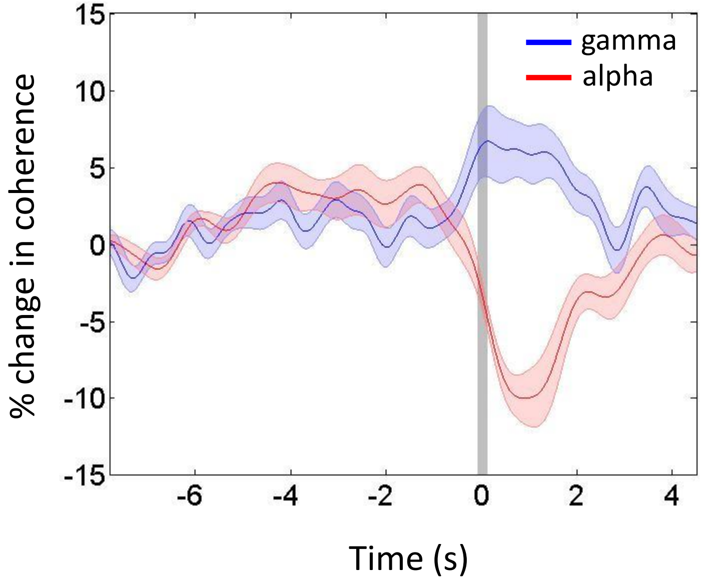

Supplementary Figure 2

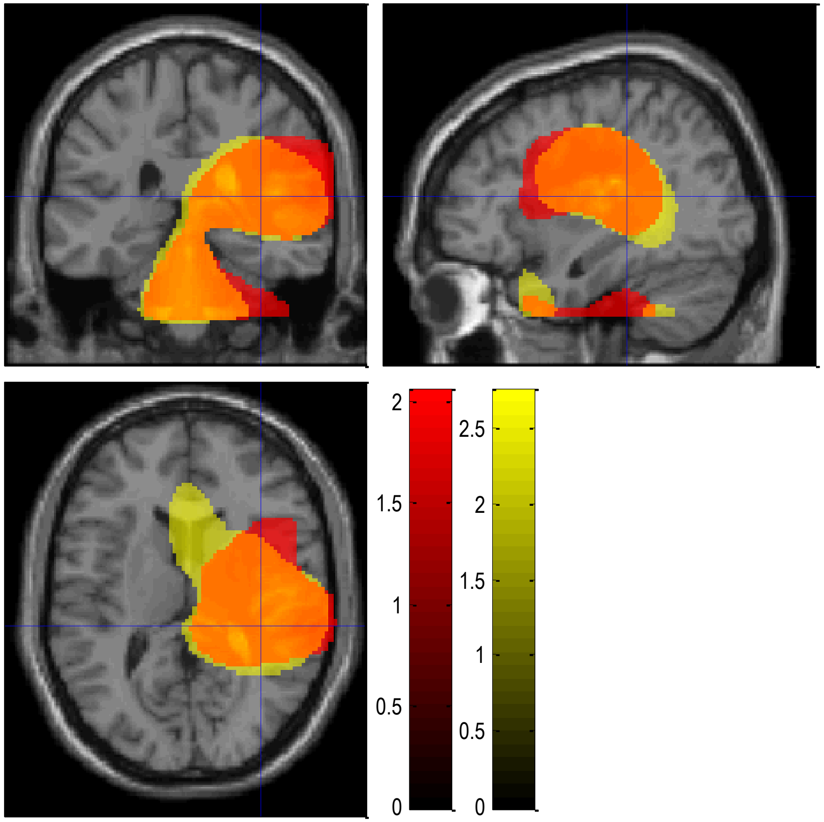

Supplementary Figure 3

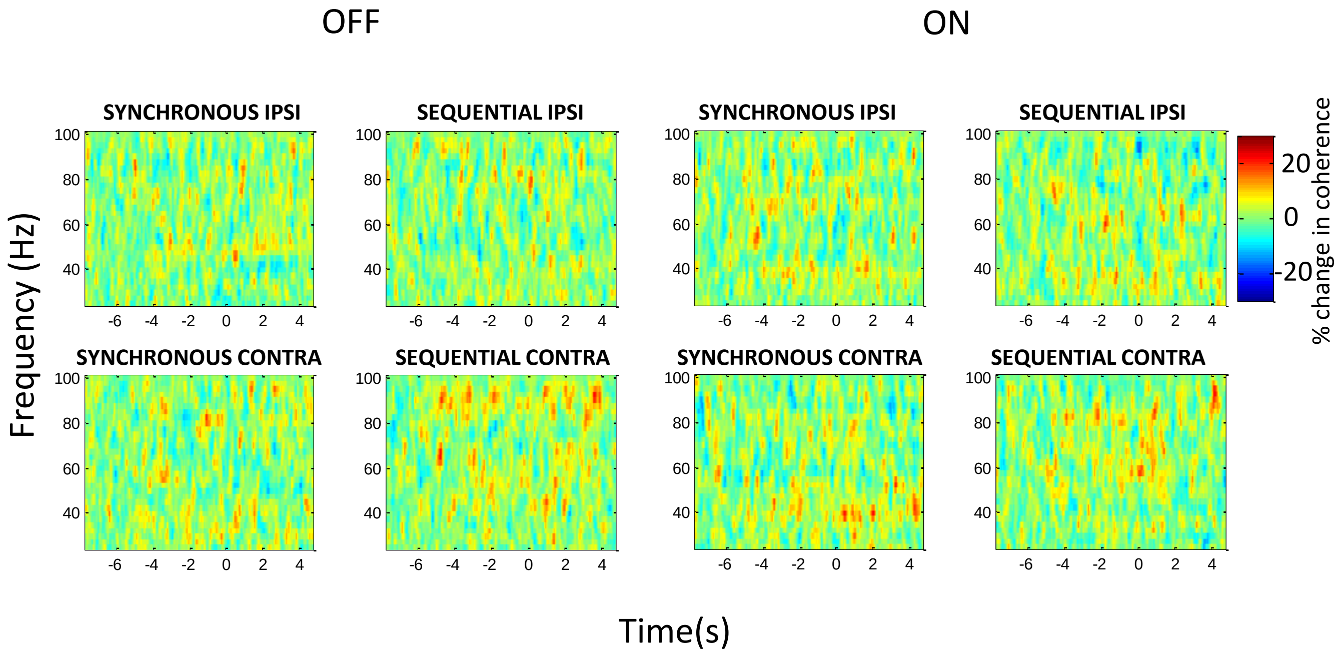

Supplementary Figure 4

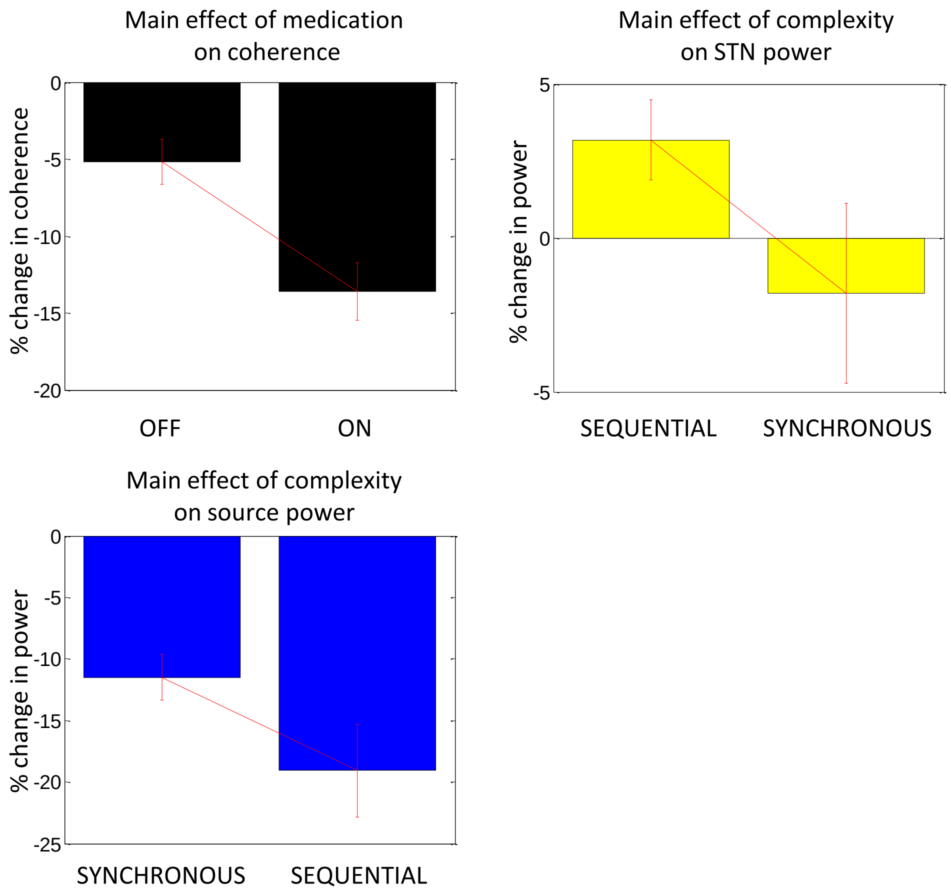

Supplementary Figure 5
